# Guidelines for Assessment of Gait and Reference Values for Spatiotemporal Gait Parameters in Older Adults: The Biomathics and Canadian Gait Consortiums Initiative

**DOI:** 10.3389/fnhum.2017.00353

**Published:** 2017-08-03

**Authors:** Olivier Beauchet, Gilles Allali, Harmehr Sekhon, Joe Verghese, Sylvie Guilain, Jean-Paul Steinmetz, Reto W. Kressig, John M. Barden, Tony Szturm, Cyrille P. Launay, Sébastien Grenier, Louis Bherer, Teresa Liu-Ambrose, Vicky L. Chester, Michele L. Callisaya, Velandai Srikanth, Guillaume Léonard, Anne-Marie De Cock, Ryuichi Sawa, Gustavo Duque, Richard Camicioli, Jorunn L. Helbostad

**Affiliations:** ^1^Department of Medicine, Division of Geriatric Medicine, Sir Mortimer B. Davis—Jewish General Hospital and Lady Davis Institute for Medical Research, McGill University Montreal, QC, Canada; ^2^Dr. Joseph Kaufmann Chair in Geriatric Medicine, Faculty of Medicine, McGill University Montreal, QC, Canada; ^3^Centre of Excellence on Aging and Chronic Diseases of McGill Integrated University Health Network QC, Canada; ^4^Department of Neurology, Geneva University Hospital and University of Geneva Geneva, Switzerland; ^5^Division of Cognitive & Motor Aging, Department of Neurology, Albert Einstein College of Medicine, Yeshiva University Bronx, NY, United States; ^6^Geriatric Department, Liège University Hospital Liege, Belgium; ^7^Laboratory of Human Motion Analysis, Liège University Liege, Belgium; ^8^Centre for Memory and Mobility Luxembourg City, Luxembourg; ^9^Basel University Center for Medicine of Aging, Felix Platter Hospital and University of Basel Basel, Switzerland; ^10^Faculty of Kinesiology and Health Studies, Neuromechanical Research Centre, University of Regina Regina, SK, Canada; ^11^Department of Physical Therapy, College of Rehabilitation Sciences, University of Manitoba Winnipeg, MB, Canada; ^12^Division of Geriatrics, Angers University Hospital Angers, France; ^13^Centre de Recherche, Institut Universitaire de Gériatrie de Montréal Montreal, QC, Canada; ^14^Department of Medicine and Montreal Heart Institute, University of Montreal Montreal, Canada; ^15^Aging, Mobility and Cognitive Neuroscience Laboratory, University of British Columbia Vancouver, BC, Canada; ^16^Andrew and Marjorie McCain Human Performance Laboratory, Richard J. Currie Center, Faculty of Kinesiology, University of New Brunswick Fredericton, NB, Canada; ^17^Menzies Institute of Medical Research, University of Tasmania Hobart, TAS, Australia; ^18^Stroke and Ageing Research Group, Department of Medicine, Southern Clinical School, Monash University Melbourne, VIC, Australia; ^19^Research Center on Aging, CIUSSS de l'Estrie-CHUS Sherbrooke, QC, Canada; ^20^Department of Geriatrics and Department of Primary and Interdisciplinary Care (ELIZA), University of Antwerp and AZ St. Maarten Mechelen Antwerp, Belgium; ^21^Department of Physical Therapy, School of Health Sciences at Narita, International University of Health and Welfare Narita, Japan; ^22^Australian Institute for Musculoskeletal Science, University of Melbourne and Western Health St. Albans, VIC, Australia; ^23^Division of Neurology, Department of Medicine, University of Alberta Edmonton, AB, Canada; ^24^Department of Neuro-Medicine and Movement Science, Faculty of Medicine and Health Sciences, Norwegian University of Science and Technology Trondheim, Norway; ^25^Clinic for Clinical Services, St. Olav University Hospital Trondheim, Norway

**Keywords:** gait, aged, guidelines, reference values

## Abstract

**Background:** Gait disorders, a highly prevalent condition in older adults, are associated with several adverse health consequences. Gait analysis allows qualitative and quantitative assessments of gait that improves the understanding of mechanisms of gait disorders and the choice of interventions. This manuscript aims (1) to give consensus guidance for clinical and spatiotemporal gait analysis based on the recorded footfalls in older adults aged 65 years and over, and (2) to provide reference values for spatiotemporal gait parameters based on the recorded footfalls in healthy older adults free of cognitive impairment and multi-morbidities.

**Methods:** International experts working in a network of two different consortiums (i.e., Biomathics and Canadian Gait Consortium) participated in this initiative. First, they identified items of standardized information following the usual procedure of formulation of consensus findings. Second, they merged databases including spatiotemporal gait assessments with GAITRite® system and clinical information from the “Gait, cOgnitiOn & Decline” (GOOD) initiative and the Generation 100 (Gen 100) study. Only healthy—free of cognitive impairment and multi-morbidities (i.e., ≤ 3 therapeutics taken daily)—participants aged 65 and older were selected. Age, sex, body mass index, mean values, and coefficients of variation (CoV) of gait parameters were used for the analyses.

**Results:** Standardized systematic assessment of three categories of items, which were demographics and clinical information, and gait characteristics (clinical and spatiotemporal gait analysis based on the recorded footfalls), were selected for the proposed guidelines. Two complementary sets of items were distinguished: a minimal data set and a full data set. In addition, a total of 954 participants (mean age 72.8 ± 4.8 years, 45.8% women) were recruited to establish the reference values. Performance of spatiotemporal gait parameters based on the recorded footfalls declined with increasing age (mean values and CoV) and demonstrated sex differences (mean values).

**Conclusions:** Based on an international multicenter collaboration, we propose consensus guidelines for gait assessment and spatiotemporal gait analysis based on the recorded footfalls, and reference values for healthy older adults.

## Introduction

Gait—the medical term used to describe the human locomotor movement of walking in healthy adults—is simple in terms of execution, but complex in terms of biomechanics and motor control (Nutt et al., 1993; Zajac et al., 2002; McCann and Higginson, 2008; Dicharry, 2010; Kuo and Donelan, 2010). Gait is usually considered as a dynamic balance condition in which the body's center of gravity is maintained within a slight base of support while moving (Farley and Ferris, 1998; Dicharry, 2010; Kuo and Donelan, 2010). During the past decade, it has been highlighted that even the simplest walking condition, such as straight-line walking at a comfortable steady-state pace without any disturbance, involves important cortical networks and cognitive functions (Alexander and Crutcher, 1990; Seidler et al., 2010; Zwergal et al., 2012; Beauchet et al., 2015b, 2016).

Numerous studies show that gait changes over an individual's lifetime (Nutt et al., 1993; Hausdorff et al., 1996; Nutt, 2001; Verghese et al., 2006; Montero-Odasso et al., 2012). Although, gait disorders are common in older (i.e., >65 years) adults, they are not unavoidable. With aging, there are physiological changes in the sensorimotor systems, which when combined with adverse effects of chronic diseases, may cause gait disorders (i.e., a deviation of normal gait performance leading to gait instability and related adverse health consequences; American Geriatrics Society and British Geriatrics Society and American Academy of Orthopedic Surgeons Panel on Falls Prevention, 2001; Nutt, 2001). Gait disorders in old age are a risk factor for falls and are associated with increased morbidity, mortality, loss of independent living, disability, altered quality of life, and as such can lead to increased health care expenditures (Panel on Prevention of Falls in Older Persons and American Geriatrics Society and British Geriatrics Society, 2011). The prevalence of gait disorders can be as high as 80% in the oldest-old (i.e., >85 years) age category and gait disorders represent a major worldwide concern based on their expanding prevalence (American Geriatrics Society and British Geriatrics Society and American Academy of Orthopedic Surgeons Panel on Falls Prevention, 2001; Verghese et al., 2006; Panel on Prevention of Falls in Older Persons and American Geriatrics Society and British Geriatrics Society, 2011).

The assessment of gait characteristics in older adults has enhanced our understanding of the mechanisms of gait disorders, which have been helpful in developing preventive and curative interventions (Nutt, 2001; Panel on Prevention of Falls in Older Persons and American Geriatrics Society and British Geriatrics Society, 2011). Clinical gait assessment has typically been based on visual observation (Nutt, 2001). However, this approach has two main limitations. First, visual observation depends on the background and experience of the clinician who performs the gait assessment, which explains the poor inter-rater reliability of this approach (Eastlack et al., 1991; Kressig et al., 2006). Second, a limited amount of information is collected, which limits the possibility of detecting gait impairments at an early stage as well as understanding the disorganization of gait control (Kressig et al., 2006; Montero-Odasso et al., 2012). The use of quantitative and standardized clinical tests, such as the Timed Up & Go (TUG) test has been shown to be useful as a complement to visual gait observation (Podsiadlo and Richardson, 1991). Indeed, it improves the inter-rater reliability of gait assessment and provides a common objective language that facilitates exchanges between clinicians and researchers. However, it is insufficient in detecting relevant subtle gait abnormalities like changes in gait variability (Kressig et al., 2006; Beauchet et al., 2014a). For instance, an increase in stride time variability (STV) has been identified as the best motor phenotype of cognitive decline in older adults, suggesting that increases in STV could be used to improve the prediction of dementia such as Alzheimer Disease (AD; Montero-Odasso et al., 2012; Beauchet et al., 2014a). It has been proposed that subclinical gait changes may be used as a surrogate marker of development of future diseases or adverse clinical outcomes, such as falls or disability (Verghese et al., 2009; Rao et al., 2011; Ayers et al., 2014; Beauchet et al., 2014a; Artaud et al., 2015).

Currently, advanced technology has changed the practice of gait analysis because it surpasses the limits of clinical observation (i.e., visual observation and standardized test) of gait and is easily accessible and feasible (Webster et al., 2005; Beauchet et al., 2008). The initial trade-off between the accuracy of gait measuring systems and their clinical use due to cost, labor-intensity, and time consumption has disappeared. There are numerous validated and user-friendly portable gait analysis systems, like electronic gait mats, insole footswitch systems and body worn inertial sensor systems that allow objective gait parameters to be easily obtained at low cost (Kressig et al., 2006; Beauchet et al., 2008). Gait analysis systems may be separated into three categories: the first includes non-wearable sensors and consists of devices based on image processing and pressure-sensitive floor sensors, such as the GAITRite® system, which provided all spatiotemporal parameters based on the recorded footfalls. The second category includes wearable sensors such as pressure-sensitive insoles and body worn accelerometers/inertial measurement units (IMUs), with this last category providing the opportunity to analyse gait outside the laboratory and obtain information about gait during the individual's everyday activities. The third category of devices includes a combination of both previous systems. Though promising, the research on gait characteristics derived from wearable sensors in free living situations is still in its infancy. It is therefore too early to give strong recommendations on gait assessment and on the protocols that should be used to derive reliable and valid information about gait from these systems.

While this is an important advancement for researchers, as well as for patients and clinicians, it presents a new challenge based on a combination of different issues: (1) the lack of consensus on which gait parameters to assess and their clinical relevance; (2) the lack of a consensus concerning data acquisition; (3) the lack of standardized data from a large number of people to correctly define reference values related to healthy aging; (4) the excessive fragmentation, dispersion and confinement of data, skills, and knowledge of teams of researchers and/or clinicians; (5) and finally the lack of sufficient research funding in science and medicine. The successful future of scientific and medical research in the field of gait disorders mainly depends on sharing and/or pooling of resources, research and databases between teams. Hence, there is an emergence of networks with a common interest to provide mutual assistance and useful information. Recently, two networks have been formalized, with the aim of helping clinicians and researchers to increase their knowledge and improve the field of age-related gait disorders by sharing knowledge and data sets: these are (1) the Biomathics (Beauchet et al., 2014c) and (2) the Canadian Gait Consortium. Both consortiums connect academic research teams working on age-related gait changes, and share their databases in order to compound a larger, more comprehensive and representative database. This provides fast and comprehensive answers to research questions with minimal additional financial resources and large population-based samples. Furthermore, it is likely that some objectives identified in a specific study may be relevant to other teams, and at the very least the initial investigators can respond to queries of a secondary team. In such cases, the requesting team launches an initiative within the consortium and contacts all team members who may be able to help. Willing researchers are included in the initiative to participate in the research, contribute to the collaborative publication and be included in the list of co-authors depending on their contribution to the study and the number of included participants. For instance, the Biomathics consortium recently focused on gait disorders in older individuals with cognitive decline: the objective was to compare spatiotemporal gait parameters based on the recorded footfalls in cognitively healthy individuals (CHIs), individuals with amnestic (aMCI) and non-amnestic mild cognitive impairment (naMCI), and individuals with mild and moderate stages AD and non-Alzheimer's disease (non-AD; Allali et al., 2016). They merged databases for a first initiative called “Gait, cOgnitiOn & Decline” (GOOD), which involved 2717 participants and represented the largest database in this field of research. The GOOD study demonstrated that spatiotemporal gait parameters are more disturbed in the advanced stages of dementia with worse performance in the non-AD dementias than in AD. These results suggest that quantitative gait parameters may be used for improving the accuracy of classifying dementia (Allali et al., 2016), as well as supporting clinical follow-ups that try to prevent adverse events such as falls or disability.

This first initiative underscored the requirement of utilizing standardized assessment when performing spatiotemporal gait analysis. Although, some reference values for gait parameters in older adults already exist (Oberg et al., 1993; Oh-Park et al., 2010; Bohannon and Williams Andrews, 2011; Hollman et al., 2011; Hass et al., 2012), this first initiative demonstrated that there is a need for quantitative reference values of spatiotemporal gait parameters for large numbers of healthy older adults. Importantly, older adults are considered to be healthy when they are free of cognitive deficits and comorbidities. Combining and integrating evaluations performed in populations from different countries is crucial for the development of future research on gait disorders. Indeed, the definition of gait disorders requires comparisons with quantitative reference values for spatiotemporal gait parameters in healthy older adults with diverse social, cultural, ethnic, and demographic backgrounds. Based on this first experience of the GOOD initiative, the Biomathics and Canadian Gait Consortiums decided to launch an initiative with the following aims: (1) to give consensus guidance for clinical and spatiotemporal gait analysis based on the recorded footfalls in older adults aged 65 years and over, and (2) to provide reference values for spatiotemporal gait parameters based on the recorded footfalls in healthy older (i.e., >65 years) adults free of cognitive impairment and multi-morbidities.

## Methods

### Guidelines for clinical and spatiotemporal gait analysis based on the recorded footfalls in older adults aged 65 years and over

The guidelines for clinical and spatiotemporal gait analysis based on the recorded footfalls in older adults followed the usual procedure of formulation of a consensus finding, consisting of a three-step process (Annweiler et al., 2015). In the first step, between May and October 2015, the lead author (OB) invited members of the Biomathics and Canadian Gait Consortiums composed with experts of gait disorders in aging, to form a group. The members of both consortiums are experts in gait and/or movement and are presented in Table 1. In a second step from July 2015 to May 2016, all experts communicated by email, phone calls or videoconferencing with the first author to identify items required for spatiotemporal gait analysis in older adults. The first author, as the leader of both consortiums, contacted each member to explain the initiative, obtain their agreement to the consensus procedures, and propose an initial version of the guidelines. Each member of the consortium formulated changes and/or proposed additional information. The first author merged all changes and wrote the second version of the guidelines. All experts reviewed this version and finally a consensual agreement was obtained. A dataset of common items divided into three categories was selected: demographic characteristics, clinical characteristics, and gait characteristics. Furthermore, a standardized procedure for spatiotemporal gait analysis based on the recorded footfalls was defined and two types of datasets were individualized: a minimum dataset corresponding to items required for all gait analysis in older individuals, and a full dataset corresponding to items of the minimum dataset plus additional items recorded when possible and for specific purposes. All selected items are shown in Table 2.

**Table 1:** Composition of Biomathics and Canadian Gait Consortiums.

**Country/Canadian province**	**Town**	**University**	**Centre**	**Reference person**
**BIOMATHICS CONSORTIUM**				
Australia	Hobart	University of Tasmania	Menzies Institute of Medical Research	Michele L Callisaya; PhD
	Melbourne	University of Melbourne & Western Health	Australian Institute for Musculoskeletal Science	Gustavo Duque; MD, PhD
	Victoria	Monash University	Department of Medicine	Velandai Srikanth; PhD
Belgium	Antwerp	University of Antwerp	Department of geriatrics and department of primary and interdisciplinary care (ELIZA)	Anne-Marie De Cock; MD
	Liege	University of Liege	Department of Geriatrics	Sylvie Gilain; MD
France	Angers	University of Angers	Department of Neuroscience, Geriatrics division	Cyrille P Launay; MD, PhD
Japan	Chiba-ken	University of Health and Welfare	Department of Physical Therapy, School of Health Sciences at Narita International	Ryuichi Sawa; PhD
Luxembourg	Luxembourg-city	Zitha Senior	Centre for Memory and Mobility	Jean-Paul Steinmetz; PhD
Norway	Trondheim	Norwegian University of Science and Technology	Department of Neuromedicine and Movement Science	Jorunn L. Helbostad; PT, PhD
USA	New York	Yeshiva University	Department of Neurology, Division of Cognitive & Motor Aging	Joe Verghese; MD, MBBS
Switzerland	Basel	University of Basel	Basel University Center for Medicine of Aging	Reto W. Kressig; MD
	Geneva	University of Geneva	Department of Neurology	Gilles Allali; MD, PhD
**CANADIAN GAIT CONSORTIUM**				
Alberta	Edmonton	University of Alberta	Department of Medicine, Division of Neurology	Richard Camicioli; MD, PhD
British Columbia	Vancouver	University of British Columbia	Aging, Mobility, and Cognitive Neuroscience Lab Djavad Mowafaghian Centre for Brain Health	Teresa Liu-Ambrose; PT, PhD
Manitoba	Winnipeg	University of Manitoba	College of Rehabilitation Sciences	Tony Szturm; PT, PhD
Quebec	Montreal	University of Concordia	Perform institute	Louis Bherer; PhD
		University of McGill	Department of Medicine, Division of Geriatrics, Jewish General Hospital	Olivier Beauchet; MD, PhD
		University of Montreal	Institut universitaire de gériatrie Montreal Heart Institute Research Center and Departement of Medicine	Sébastien Grenier; PhD Louis Bherer; PhD
	Sherbrooke	University of Sherbrooke	Research Centre on Aging	Léonard Guillaume; PhD
New Brunswick	Fredericton	University of New Brunswick	Richard J. Currie Center	Victoria L. Chester; PhD
Saskatchewan	Regina	University of Regina	Neuromechanical Research Centre, Faculty of Kinesiology and Health Studies	John M. Barden; PhD

**Table 2:** Selected items for gait analysis in the elderly.

**Items for the minimum dataset**	**Additional items for the full dataset**
**DEMOGRAPHIC CHARACTERISTICS**	
Age (year)	
Sex	
Ethnicity coded as follows: 1, Black; 2, Caucasian; 3, Asian; 4, Other	
**CLINICAL CHARACTERISTICS**	
Height (m)	
Weight (kg)	
Medication; Number of therapeutic classes used per day >3 (coded yes vs. no)	
	Number of therapeutic classes taken daily
	Use of psychoactive drugs (i.e., benzodiazepines, antidepressants, neuroleptics) (coded yes vs. no)
History of falls (i.e., defined as an event resulting in a person coming to rest unintentionally on the ground or at another lower level, not as the result of a major intrinsic event or an overwhelming hazard) in the previous 12-month period (coded yes vs. no)	Recurrent falls (i.e., >2) (coded yes vs. no) Severe falls (i.e., fractures, cranial trauma, large and/or deep skin lesions, post-fall syndrome; inability to get up; time on ground >1 h; hospitalization) (coded yes vs. no).
	Fear of falling (Are you afraid of falling? Never, almost never, sometimes, often, and very often)
Neurological diseases:	
• Dementia (coded yes vs. no)	• Cognitive complaint (coded yes vs. no)
	• Mild cognitive impairment (coded yes vs. no)
	• Dementia (coded yes vs. no), if yes stage (i.e., mild, moderate, severe) and etiology (i.e., AD, non-AD neurodegenerative, non-AD vascular, mixed)
	• Global cognitive performance: MoCA score (Nasreddine et al., 2005)
• Other (coded yes vs. no)	• Parkinson's disease or parkinsonian syndromes (coded yes vs. no)
	• Idiopathic normal pressure hydrocephalus (coded yes vs. no)
	• Cerebellar disease (coded yes vs. no)
	• Myelopathy (coded yes vs. no)
	• Peripheral neuropathy (coded yes vs. no)
Depressive symptoms (coded yes vs. no)	4-item Geriatric Depression Scale score (Shah et al., 1997)
Anxiety symptoms (coded yes vs. no)	5-item Geriatric Anxiety Inventory (Byrne and Pachana, 2011)
Major orthopedic diagnoses (e.g., osteoarthritis) involving the lumbar vertebrae, pelvis or lower extremities (coded yes vs. no)	
Vision disorders (coded yes vs. no)	Distance binocular vision measured at 5 m with a standard scale, vision assessed with corrective lenses if needed
Lower limb proprioception disorders (coded yes vs. no)	Lower limb proprioception evaluated with a graduated tuning fork placed on the tibial tuberosity: The mean value obtained for the left and right sides (/8)
Muscle strength impairment (coded yes vs. no)	Hand grip strength: mean value of the highest value of maximal isometric voluntary contractions (3 trials) measured with computerized dynamometers expressed in Newtons per square meter
Use of walking aid (coded yes vs. no)	
**GAIT CHARACTERISTICS**	
**Clinical analysis**	
Subjective self-reported difficulties (coded never, almost never, sometimes, often, and very often)	
Clinical gait abnormalities (coded yes vs. no)	
Timed Up & Go score (s) (Podsiadlo and Richardson, 1991)	Timed Up & Go imagined form score (s) (Beauchet et al., 2010)
Walking speed: time to walk 4 m at steady-state walking	
**Spatiotemporal analysis**	
Conditions ✓ In a quiet, well-lit environment ✓ Steady state walking (acceleration and deceleration phase of 1 m each) ✓ Wearing participant's own footwear ✓ Usual self-selected walking speed	
	Fast walking speed Dual tasking: ✓ Backward counting by ones from 50 ✓ Verbal fluency task (animal names)
Parameters Walking speed [mean value (cm/s)] ✓ Stride time [mean value (ms) and coefficient of variation (%)] ✓ Swing time [mean value (ms) and coefficient of variation (%)] ✓ Stride width [mean value (cm) and coefficient of variation (%)]	
	Stride length [mean value (cm) and coefficient of variation (%)] Stance time [mean value (ms) and coefficient of variation (%)] Single support time [mean value (ms) and coefficient of variation (%)] Double support time [mean value (ms) and coefficient of variation (%)] Stride velocity [mean value (cm/s) and coefficient of variation (%)]

*m, meter; kg, kilogram; s, second; cm, centimeter*.

### Quantitative reference values for spatiotemporal gait parameters based on the recorded footfalls

#### Participant selection

Data were extracted from two databases: the GOOD initiative (Clinical trials registration number: NCT02350270) (Allali et al., 2016) and the Generation 100 (Clinical trials registration number: NCT01666340) (Stensvold et al., 2015). The GOOD initiative was based on a cross-sectional design such that the main objective was to compare spatiotemporal gait characteristics based on the recorded footfalls of CHIs, and participants with MCI or dementia. Data collection, study procedures and criteria for categorization of participants have been described in detail elsewhere (Allali et al., 2016). In brief, data from seven countries (Australia, Belgium, France, India, Luxembourg, Switzerland, and the United States) were merged. Data sources were the “Tasmanian Study of Cognition and Gait” (TASCOG) (Tasmanian), the Mechelen memory clinic database (Belgium), the “Gait and Alzheimer Interactions Tracking” (GAIT) study (France), the “Kerala-Einstein Study” (KES) (India), the Center for Memory and Mobility (Luxembourg), the “Central Control of Mobility in Aging” (US), and the Basel mobility center (Switzerland).

The Generation 100 study is a population-based large randomized controlled clinical trial (Stensvold et al., 2015). The primary aim of this study is to examine the effects of 5 years of exercise training on mortality in the elderly (Stensvold et al., 2015). The data collection and study procedures have been described in detail elsewhere (Stensvold et al., 2015). In summary, it is an ongoing phase IIb clinical trial. The participants are stratified by sex and marital status and randomized 1:1 into an exercise training group or a control group. They are assessed at baseline and at follow-up after 1, 3, and 5 years. For this analysis, we used the data collected at baseline.

Exclusion criteria for the present study were age <65 years, non-Caucasian, cognitive decline (i.e., MCI and dementia), walking with personal assistance, polypharmacy defined as more than 3 therapeutic drug classes taken daily, history of falls in the past 12-month period, the presence of depressive and/or anxiety symptoms, moderate or severe distance vision impairment (when information was accessible), and absence of spatiotemporal gait data. From the 2,717 participants initially recruited in the GOOD initiative, 548 (20.2%) healthy older adults met the inclusion criteria. A total of 457 (29.7%) participants from the 1,541 participants who had a gait assessment at baseline in the Generation 100 study met the inclusion criteria. Fifty-one of the 1005 (19.7%) identified participants were excluded because of incomplete gait data. Finally, 954 participants were included in the analysis.

#### Assessment

Age, sex, and anthropometric measures (i.e., height in metres and weight in kilograms) were recorded. Body mass index (BMI, in kg/m^2^) was also calculated. Spatiotemporal gait parameters based on the recorded footfalls were measured during steady-state walking using the GAITRite®-system. This gait system is an electronic walkway with an integrated pressure-sensitive electronic surface connected to a portable computer via an interface cable. The GAITRite®-system is a well-established method of quantifying gait and provides reliable and accurate measures of spatiotemporal gait parameters. Spatiotemporal gait parameters have shown excellent test-retest reliability in clinical and research settings in community-dwelling older people when using the GAITRite®-system (Brach et al., 2008). During the past decade over 100 manuscripts have been published using data collected and processed with the GAITRite® system.

The active recording area of the gait mats ranged from 4.6 m (TASCOG study) to 7.9 m (GAIT study). Participants completed one (GAIT, CCMA, and KES studies; the Mechelen memory clinic, the Centre for Memory and Mobility of Luxembourg-city, The Basel mobility center), two (Generation 100 study) or six (TASCOG study) trials at their usual self-selected walking speed in a quiet, well-lit environment, wearing their own footwear. The mean of the 2 (the Generation 100) or 6 trials (the TASCOG studies) was used to calculate the gait variables. The mean value and coefficient of variation [CoV = (standard deviation/mean) × 100] of the spatiotemporal gait parameters were used as outcomes. For a list of the included spatiotemporal variables, see Table 2.

#### Standard protocol approvals and registrations

Each site involved in this study obtained approval from their local ethics committee to conduct site-specific assessments: the Southern Tasmanian Health and Medical Human Research Ethics Committee for the TASCOG study (Australia), the ethics committee of Angers University hospital for the GAIT study (France), the ethics committee of Emmaus—St Maarten General Hospital Mechelen for the Mechelen memory clinic database (Belgium), the institutional ethics committee of Baby Memorial Hospital for KES study (India), the ethics committee of Luxembourg for the Center for Memory and Mobility database (Luxembourg), the ethics committee of Albert Einstein College of Medicine for the “Central Control of Mobility in Aging” (US) study, and the ethics committee of Basel for the Basel mobility center database (Switzerland). The ethics committee of Angers (France) University hospital approved the GOOD initiative (2014/17). The regional committee of Mid Norway for Medical and Health Research Ethics approved the transfer and the merging (number 2015/1797) of the Generation 100 database with the GOOD database.

#### Statistics

Participants' baseline characteristics were summarized using means and standard deviations or frequencies and percentages. Participants were separated into three age groups (65–74 years, 75–84 years, and >85 years), and each group was dichotomized by sex. First, between-group comparisons were performed using unpaired *t*-test or Mann–Whitney tests, as appropriate. *P* < 0.0006 were considered as statistically significant after adjustments for multiple comparisons (*n* = 79). Second, multiple linear regressions showing the association of each spatiotemporal gait parameter (dependent variable) with age and sex (independent variable), adjusted for BMI and test center were performed. *P* < 0.05 were considered as statistically significant. All statistics were performed using SPSS (version 15.0; SPSS, Inc., Chicago, IL).

## Results

### Guidelines for clinical and spatiotemporal gait analysis based on the recorded footfalls

Two complementary sets of standardized information were identified: a minimal data set and a full data set. All items of both sets are shown in Table 2. They have been separated into three categories: demographic, clinical, and gait characteristics. This last category has been divided into clinical and spatiotemporal gait analysis based on the recorded footfalls.

#### Demographic and clinical characteristics

Demographic (i.e., age in years, sex and ethnicity) and anthropometric items [height in meters (m), weight in kilograms (kg), body mass index (BMI) in kg/m^2^], are required because each may influence spatiotemporal gait parameters (American Geriatrics Society and British Geriatrics Society and American Academy of Orthopedic Surgeons Panel on Falls Prevention, 2001; Kressig et al., 2006; Verghese et al., 2006; Beauchet et al., 2008; Dicharry, 2010; Panel on Prevention of Falls in Older Persons and American Geriatrics Society and British Geriatrics Society, 2011). Given that the burden of disease can influence gait performance, it was decided to record this information as well (American Geriatrics Society and British Geriatrics Society and American Academy of Orthopedic Surgeons Panel on Falls Prevention, 2001; Panel on Prevention of Falls in Older Persons and American Geriatrics Society and British Geriatrics Society, 2011). Different scales have been developed to score the burden of morbidity, but they remain difficult to use in older adults, especially because of possible recall bias when reporting chronic disease among individuals with cognitive disorders, and lack of feasibility in clinical practice (due to their complexity and value for physicians, physiotherapists, or other health care professionals; Linn et al., 1968; Parmelee et al., 1995; Salvi et al., 2008; de Decker et al., 2013). Recently, an independent association was found between the Cumulative Illness Rating Scale Geriatric form (CIRS-G), which provides a morbidity score, and the number of drug classes taken daily (de Decker et al., 2013). The results showed that an increase of three drug classes corresponds to a one-point increase on the CIRS-G (de Decker et al., 2013). This result is consistent with previous studies in the general population, which reported that pharmacy data using the Anatomical Therapeutic Chemical Classification (ATCC) system might be used to provide reliable prevalence estimates of several common comorbid conditions (Von Korff et al., 1992; Maio et al., 2005; Chini et al., 2011). In addition, it has been demonstrated that pharmacy data provide a stable measure of morbidity status, and are associated with physician-rated disease severity as well as with individual-rated health status (Von Korff et al., 1992). Hence, the decision was made to record the use of drugs in the clinical assessment. Polypharmacy is defined as the use of more than three drugs per day, which was used as the item for the minimum data set, and was combined with the exact number of therapeutic drug classes taken daily and the use of psychoactive drugs (i.e., benzodiazepines, antidepressants, neuroleptics), which was coded as yes or no in the full dataset.

Information about falls, with a fall being defined as an event resulting in a person coming to rest unintentionally on the ground or at another lower level, not as the result of a major intrinsic event or an overwhelming hazard, in the previous 12 month-period before the assessment, is also proposed (American Geriatrics Society and British Geriatrics Society and American Academy of Orthopedic Surgeons Panel on Falls Prevention, 2001; Panel on Prevention of Falls in Older Persons and American Geriatrics Society and British Geriatrics Society, 2011). For the minimum data set, only the existence (or not) of a fall(s) history is required, while for the full data set information on recurrence (i.e., >2 falls) and severity (defined as fractures, cranial trauma, large, and/or deep skin lesions, post-fall syndrome including an association of fear of falling (FOF), postural instability with absence of postural reflexes, inability to get up, time on ground >1 h, and hospitalization) are proposed for the data collection. Recently, a systematic review and meta-analysis reported that FOF might increase gait instability (Ayoubi et al., 2015). Thus, it was determined to measure FOF using the single question: “Are you afraid of falling?” with a graded answer (i.e., never, almost never, sometimes, often, and very often) for the full dataset.

In addition to FOF, collecting information on disorders or diseases that directly influence gait performance is also advised. First, information on neurological diseases (limited to the existence or non-existence of dementia) and other diseases (coded as yes or no) are collected for the minimal data set. Information on memory complaints, MCI, nature of dementia (i.e., AD, non-AD neurodegenerative, non-AD vascular, mixed), Parkinson disease, idiopathic normal pressure hydrocephalus, cerebellar disease, stroke, myelopathy, and peripheral neuropathy are also proposed for the full dataset (Alexander and Crutcher, 1990; Nutt et al., 1993; Nutt, 2001; Verghese et al., 2006; Montero-Odasso et al., 2012). A quantification of global cognitive functioning is also recommended, using for example the Montreal Cognitive Assessment (MoCA) (Nasreddine et al., 2005). In addition, among the neuropsychiatric disorders, it is important to collect information about depression symptoms because they can lead to gait instability and falls. This is limited to a simple binary question in the minimum data set and the score for the 4-item geriatric depression scale in the full data set (Shah et al., 1997). A measure of anxiety is also proposed using the 5-item Geriatric Anxiety Inventory (Byrne and Pachana, 2011).

Information on major orthopedic diagnoses (e.g., osteoarthritis) involving the lumbar vertebrae, pelvis, or lower extremities, coded yes vs. no, as well as the use of a walking aid, should also be recorded (American Geriatrics Society and British Geriatrics Society and American Academy of Orthopedic Surgeons Panel on Falls Prevention, 2001; Panel on Prevention of Falls in Older Persons and American Geriatrics Society and British Geriatrics Society, 2011).

Information on sensory and motor subsystems such as muscle strength, lower-limb proprioception and vision are required because the age-related impairment in the performance of these subsystems may affect gait performance (Beauchet et al., 2014a). For the minimal data set, impairments were coded as binary (i.e., yes or no), while in the full dataset standardized measures are required. First, the Maximum isometric Voluntary Contraction (MVC) of handgrip strength must be measured with a computerized hydraulic dynamometer. The test should be performed three times with the dominant hand. The mean value of MVC over the three trials should be used as the outcome measure. Second, distance binocular vision should be measured at a distance of 5 m with a standard scale (Lord et al., 1994). Vision needs to be assessed with corrective lenses, if used regularly. Third, lower extremity vibration sense should be measured, using a graded tuning fork placed on a bony area, such as the tibial tuberosity, medial malleolus or big toe. This is correlated with proprioception, which is critical to balance (Beauchet et al., 2014a).

#### Gait characteristics

Before conducting a spatiotemporal gait analysis based on the recorded footfalls, a standardized clinical evaluation is advised. First, the individual's subjective perception of gait difficulties is registered using a single question: “Do you have any difficulty walking?” with a graduated answer (i.e., never, almost never, sometimes, often, and very often). Second, a visual observation of gait during habitual walking is proposed with a binary answer (yes vs. no) to the question “are there gait abnormalities during physical examination?”

Third, the TUG test score and gait speed (distance divided by ambulation time) when walking a distance of 4 m at a steady-state pace is suggested (Podsiadlo and Richardson, 1991; Goldberg and Schepens, 2011). These measures are proposed for the minimal dataset, while for the full data set an additional measure is proposed; that being the time to achieve the imagined TUG (iTUG) (Beauchet et al., 2015a). Exploring the higher levels of gait control may be more difficult in clinical practice. There are two alternatives: using a dual-task paradigm (i.e., walking while simultaneously executing an attention-demanding task), or using motor imagery of gait (i.e., the mental simulation of gait without its actual execution; Beauchet et al., 2015a). Recently, interest in the latter alternative has been underscored using the mental chronometry approach applied to the TUG, a well-known motor test used in clinical practice (Beauchet et al., 2010, 2014b, 2015a). The TUG is a standardized assessment of a basic functional mobility task of relevance to daily living and records the time needed to stand up, to walk 3 m, to turn back and sit down (Podsiadlo and Richardson, 1991). It has been reported that cognitive performance, and in particular executive functioning, contributes to the temporal correspondence between executing and imaging gait in individuals with neuropsychiatric conditions like dementia, schizophrenia or multiple sclerosis (Linn et al., 1968; Von Korff et al., 1992; Oberg et al., 1993; Lord et al., 1994; Parmelee et al., 1995; Shah et al., 1997; Maio et al., 2005; Nasreddine et al., 2005; Brach et al., 2008; Salvi et al., 2008; Beauchet et al., 2010, 2014a,b, 2015a; Bohannon and Williams Andrews, 2011; Byrne and Pachana, 2011; Chini et al., 2011; Goldberg and Schepens, 2011; Allali et al., 2012; Hass et al., 2012; Lallart et al., 2012; de Decker et al., 2013; Annweiler et al., 2015; Ayoubi et al., 2015; Stensvold et al., 2015). It has also been shown that older individuals with cognitive impairment executed the iTUG more rapidly than they performed it (Allali et al., 2012; Beauchet et al., 2015a). On the contrary, there has been no significant difference between the two conditions in healthy younger adults (Lallart et al., 2012). This difference in terms of performance between pTUG and iTUG, called “delta TUG,” can be interpreted as the awareness of movement and physical performance, and thus may be used as a biomarker of the disorders of higher levels of gait control (Beauchet et al., 2010, 2014b, 2015a; Allali et al., 2012; Lallart et al., 2012).

It is necessary to underscore that the spatiotemporal gait analysis based on the recorded footfalls should be performed in a reproducible, quiet, well-lit environment, with patients wearing their own footwear (walking shoes, no slippers) with heel height not exceeding 3 cm and comfortable and non-restrictive clothing. Depending on the participant's fall risk, the use of safety support systems is recommended, such as a safety belt around the participant's waist. We recommend assessing the normal walking condition for the minimal data set, and for the full dataset we recommend three additional walking conditions; a fast walk at a maximum speed, and two dual-task conditions, in which the patient is instructed to walk normally while (a) counting backwards by ones starting from 50 and (b) to enumerate animal names (Kressig et al., 2006; Beauchet et al., 2012; Montero-Odasso et al., 2012). For the dual task condition, no prioritization should be given to a single task and the trial should be performed to the best of the participant's ability. Steady-state gait and gait trials in the same walking direction are required for all conditions and may be achieved by instructing participants to start walking at least 1 m prior to the data recording zone and stopping at least 1 m beyond it. It is also advisable to use simple, clear and standardized walking instructions to explain the various tasks to the participants.

Regardless of the type of category of devices used to assess gait, we recommend using a validated system that provides reliable measures. For the minimum data set, four gait parameters during normal walking including the mean value of walking speed, and mean values and coefficient of variation of stride time, swing time and stride width need to be reported. We suggest adding more stressful walking conditions (i.e., fast speed and dual tasking conditions) and reporting mean values and coefficients of variation of stride length, stance time, single and double support, and stride velocity for the full dataset. This choice is based on the fact that in terms of control of gait, gait variability has been identified as a biomarker for cortical control of gait in normal aging individuals and in individuals with dementia (Beauchet et al., 2010, 2012, 2014b, 2015a; Allali et al., 2012; Lallart et al., 2012). In addition, higher (i.e., worse) STV during normal walking has been associated with lower cognitive performance in non-demented older community-dwellers (Beauchet et al., 2012). This result has been confirmed by a meta-analysis underscoring that higher STV during normal walking was related to both MCI and dementia (Beauchet et al., 2014a). In terms of gait variability, a certain level of “healthy” variability of the motor control system is necessary to adapt to unexpected instability. Indeed, both high and low gait variability during habitual walking have been reported in younger and older CHIs with safe gait, depending on the type of gait parameters being examined (Beauchet et al., 2009). In particular, safe gait has been characterized by a low STV, an intermediate swing time variability and a high stride width variability in CHIs (Beauchet et al., 2009). These results can be explained by the fact that temporal and spatial gait parameters appear to reflect different constructs of gait control (Gabell and Nayak, 1984; Newell and Corcos, 1993; Nutt et al., 1993; Nutt, 2001; Launay et al., 2013). Stride time and stride width variability provide an indication of control of the rhythmic stepping mechanism and dynamic postural control, respectively, while swing time is indicative of both mechanisms (Gabell and Nayak, 1984; Beauchet et al., 2009). Furthermore, it is important to consider the number of steps recorded. Indeed, the accuracy of gait variability measures are highly dependent on obtaining a sufficient number of steps, with a study suggesting that a minimum of 400 steps are needed to obtain valid measures of gait variability during treadmill walking (Faude et al., 2012). However, even if it is recommended to have the highest number of gait cycles possible from a practical standpoint to assess gait variability of spatiotemporal parameters, it has been suggested that a minimum of three consecutive gait cycles should be obtained for both the left and right sides (i.e., a total of six gait cycles; Kressig et al., 2006). Furthermore, including steps from several shorter walks is recommended when obtaining the number of steps over a long walking distance is not possible.

For the collection of gait data, we suggest that gait should be assessed without assistive devices whenever possible. When a device is required it is important to describe the type of device used by the individual. Given that there are no established reference values for assistive devices, the first assessment should be used as the reference point for individuals who repeatedly use the same device.

The operational definitions of spatiotemporal gait parameters, based on GAITRite® software are as follows: (1) Stride length (in cm): anterior-posterior distance between the heel strikes of two successive placements of the same foot; stride width (in cm): lateral distance between the midlines of the right and left heels; stride time (in ms): time elapsed from the first contact of two consecutive footsteps of the same foot; swing time (in ms): time elapsed from the last contact of the current footstep to the first contact of the next footstep on the same foot; stance time (in ms): time elapsed from the initial contact and the last contact of consecutive footstep of the same foot; single support time (in ms): time elapsed from the last contact of the opposite footfall to the initial contact of the next footstep of the same foot; double support time (in ms): time elapsed during which both feet are in ground contact; stride velocity (in cm/s): stride length divided by the stride time; and walking speed (in cm/s): distance walked divided by the ambulation time.

#### Procedure for clinical and spatiotemporal gait analysis based on the recorded footfalls

All adults aged 65 and over should be systematically interviewed or examined for gait disorders at least once per year. In addition, those who report a fall or have an acute medical condition should be asked about difficulties with gait and should be examined for gait disorders.

Clinical assessment should be separated into two main parts: global and analytic clinical assessment. The global assessment detecting gait difficulties begins with watching individuals as they walk into the examination room. The use of a walking aid and its nature (i.e., cane, walker, personal assistance, and supervision) should be noticed and the individual should be asked about his/her subjective perception of gait difficulties. This visual observation should be completed with one of the two standardized motor tests to provide an objective measure of gait performance: the TUG score and the gait speed value. After this clinical assessment and if an abnormality is recorded, a spatiotemporal gait analysis based on the recorded footfalls (collection of all information described in Table 2) in laboratory setting is suggested. If necessary and based on abnormalities recorded during the clinical and clinical and spatiotemporal gait analysis, an analysis outside the laboratory using wearable sensors may be propose to obtain information about gait during the individual's everyday activities. The role of other laboratory testing and diagnostic evaluation for gait and balance disorders has not been well-studied, and there is no recommended systematic investigation to perform. However, the following complementary investigations are recommended: (1) Bone radiography in the event of acute pain, joint deformation and/or functional disability, (2) Standard 12-lead ECG in case of dizziness, 3) Blood glucose level in patients with diabetes, and (4) Serum 25OHD concentration if there is no vitamin D supplementation. Cerebral imaging in the absence of specific indications based on a clinical examination may not be necessary.

### Quantitative reference values for spatiotemporal gait parameters

Table 3 shows the group mean values, standard deviations and CoV of spatiotemporal gait parameters separated by age groups and sex. In most cases, men demonstrated greater performance for mean values (i.e., less difference relative to normal values for healthy young adults) than women, but not for CoV. This effect was observed in the total sample as well as for the 65–74 year age category. Interestingly, walking speed and stride velocity were similar in both males and females when considering the total sample and each age strata separately.

**Table 3:** Quantitative reference values (i.e., mean ± standard deviation) for spatiotemporal gait parameters by age group (65–74 years, 75–84 years and > 85 years) and sex (*n* = 954).

	**Total population (*n* = 954)**			*P*-value^*^	**Age**											
	**Total**	**Female (*n* = 437)**	**Male (*n* = 517)**		**65–74 years (*n* = 711)**			*P*-value^*^	**75–84 years (*n* = 207)**			*P*-value^*^	**>85 years (*n* = 36)**			*P*-value^*^
					**Total**	**Female (*n* = 312)**	**Male (*n* = 399)**		**Total**	**Female (*n* = 101)**	**Male (*n* = 106)**		**Total**	**Female (*n* = 24)**	**Male (*n* = 12)**	
Age (years), mean ± *SD*	72.8 ± 4.8	73.2 ± 5.1	72.4 ± 4.5	0.006	70.6 ± 2.4	70.7 ± 2.4	70.5 ± 2.3	0.649	77.6 ± 2.6	77.8 ± 2.5	77.4 ± 2.6	0.274	87.7 ± 2.8	87.2 ± 2.0	88.6 ± 4.0	0.585
BMI (kg/m^2^), mean ± *SD*	26.2 ± 4.1	26.0 ± 4.8	26.4 ± 3.3	0.105	26.0 ± 3.8	25.6 ± 4.4	26.2 ± 3.2	0.094	26.6 ± 4.1	26.2 ± 4.7	27.0 ± 3.4	0.171	28.0 ± 7.2	28.2 ± 8.4	27.6 ± 3.8	0.379
**STRIDE TIME**																
Mean value (ms)	1123.7 ± 122.4	1095.5 ± 109.8	1147.6 ± 127.4	**<0.001**	1118.5 ± 122.3	1081.5 ± 104.3	1147.3 ± 127.5	**<0.001**	1132.7 ± 117.0	1124.3 ± 109.4	1140.7 ± 123.7	0.314	1176.1 ± 140.9	1155.7 ± 139.2	1216.9 ± 141.1	0.177
CoV (%)	2.2 ± 1.1	2.2 ± 1.1	2.1 ± 1.0	0.244	2.1 ± 1.1	2.1 ± 1.0	2.1 ± 1.0	0.520	2.3 ± 1.1	2.4 ± 1.1	2.2 ± 1.0	0.053	2.8 ± 1.3	3.1 ± 1.3	2.3 ± 1.3	0.067
**SWING TIME**																
Mean value (ms)	414.1 ± 40.2	402.1 ± 36.5	424.2 ± 40.5	**<0.001**	416.3 ± 40.0	403.4 ± 36.2	426.3 ± 40.0	**<0.001**	409.6 ± 39.6	401.2 ± 36.7	417.5 ± 40.8	0.003	396.7 ± 43.1	388.6 ± 37.5	413.1 ± 50.2	0.188
CoV (%)	4.2 ± 1.8	4.2 ± 2.0	4.2 ± 1.6	0.863	4.0 ± 1.7	3.9 ± 1.9	4.1 ± 1.6	0.063	4.5 ± 1.7	4.7 ± 1.8	4.4 ± 1.6	0.199	6.0 ± 2.7	6.5 ± 2.7	4.9 ± 2.3	0.020
**STANCE TIME**																
Mean value (ms)	706.6 ± 91.2	689.3 ± 87.3	721.2 ± 92.0	**<0.001**	700.9 ± 88.1	677.6 ± 79.0	719.0 ± 90.6	**<0.001**	713.5 ± 91.6	706.6 ± 90.3	720.1 ± 92.7	0.291	779.4 ± 114.9	767.0 ± 122.6	804.0 ± 97.9	0.212
CoV (%)	3.1 ± 1.4	3.1 ± 1.3	3.1 ± 1.4	0.309	3.1 ± 1.3	3.1 ± 1.3	3.1 ± 1.4	0.743	3.2 ± 1.5	3.3 ± 1.4	3.0 ± 1.5	0.124	3.5 ± 1.7	3.8 ± 1.8	2.9 ± 1.4	0.029
**SINGLE SUPPORT TIME**																
Mean value (ms)	414.3 ± 39.8	401.5 ± 35.5	425.2 ± 40.0	**<0.001**	417.2 ± 39.5	403.4 ± 36.2	427.4 ± 39.6	**<0.001**	407.8 ± 38.7	396.8 ± 34.3	418.3 ± 39.8	**<0.001**	396.7 ± 43.1	388.6 ± 37.5	413.1 ± 50.2	0.188
CoV (%)	4.0 ± 1.8	4.1 ± 2.0	4.0 ± 1.6	0.453	3.9 ± 1.7	3.9 ± 1.9	3.9 ± 1.5	0.154	4.3 ± 1.7	4.5 ± 1.8	4.2 ± 1.6	0.102	6.0 ± 2.7	6.5 ± 2.8	4.9 ± 2.3	0.062
**DOUBLE SUPPORT TIME**																
Mean value (ms)	292.6 ± 71.0	288.1 ± 74.1	296.4 ± 68.2	0.072	284.2 ± 64.5	274.5 ± 62.1	291.8 ± 65.4	**<0.001**	305.7 ± 74.2	308.8 ± 77.3	302.9 ± 71.4	0.569	381.4 ± 100.2	376.4 ± 115.3	391.3 ± 63.1	0.398
CoV (%)	6.6 ± 2.8	6.8 ± 2.7	6.5 ± 2.8	0.079	6.8 ± 2.9	7.0 ± 2.8	6.6 ± 2.9	0.117	6.3 ± 2.5	6.5 ± 2.8	6.1 ± 2.2	0.273	6.0 ± 2.1	6.2 ± 2.8	5.5 ± 2.6	0.177
**STRIDE LENGTH**																
Mean value (cm)	134.1 ± 18.9	126.5 ± 17.1	140.7 ± 18.0	**<0.001**	138.0 ± 16.6	131.1 ± 14.8	143.3 ± 15.9	**<0.001**	126.5 ± 19.7	118.2 ± 15.1	134.4 ± 20.4	**<0.001**	102.9 ± 15.3	100.7 ± 16.2	107.3 ± 13.0	0.166
CoV (%)	2.3 ± 1.2	2.7 ± 1.3	2.2 ± 1.1	0.005	2.2 ± 1.1	2.2 ± 1.2	2.1 ± 1.0	0.087	2.6 ± 1.3	2.7 ± 1.3	2.6 ± 1.3	0.743	3.6 ± 2.1	4.1 ± 2.4	2.7 ± 1.0	0.026
**STRIDE WIDTH**																
Mean value (cm)	9.9 ± 3.1	9.4 ± 33.1	10.2 ± 3.0	**<0.001**	9.9 ± 3.1	9.5 ± 3.1	10.3 ± 3.0	0.001	9.6 ± 3.2	9.0 ± 3.4	10.1 ± 2.9	0.010	10.0 ± 3.2	9.9 ± 2.5	10.2 ± 4.3	0.804
CoV (%)	26.6 ± 49.0	30.9 ± 69.8	23.0 ± 17.2	0.013	24.6 ± 34.7	27.4 ± 48.5	22.5 ± 17.2	0.057	33.0 ± 82.6	43.4 ± 116.9	23.0 ± 12.8	0.075	28.2 ± 23.4	22.5 ± 9.1	39.5 ± 36.8	0.934
**WALKING SPEED (CM/S)**																
mean ± *SD*	121.5 ± 23.4	120.2 ± 23.8	122.7 ± 23.0	0.103	125.4 ± 21.7	126.1 ± 21.7	124.9 ± 21.6	0.488	113.9 ± 23.5	109.7 ± 21.3	118.0 ± 24.9	0.011	88.5 ± 17.8	88.3 ± 19.4	88.9 ± 14.9	0.934
**STRIDE VELOCITY**																
Mean value (cm/s)	119.9 ± 22.5	118.8 ± 23.2	120.8 ± 21.8	0.175	122.9 ± 21.1	123.6 ± 21.2	122.3 ± 21.0	0.426	114.8 ± 22.8	111.1 ± 22.7	118.5 ± 22.5	0.020	89.0 ± 17.8	88.9 ± 19.4	89.3 ± 15.0	0.251
CoV (%)	43.5 ± 1.7	3.5 ± 1.7	3.4 ± 1.6	0.244	3.4 ± 1.6	3.4 ± 1.7	3.4 ± 1.6	0.983	3.7 ± 1.7	3.8 ± 1.8	3.6 ± 1.6	0.280	4.2 ± 2.0	4.6 ± 2.0	3.5 ± 1.9	0.084

SD, standard deviation; m, meter; s, second; ms, millisecond; CoV, coefficient of variation;

* *Comparison based on unpaired t-test; P significant (i.e., P < 0.0006) indicated in bold*.

The results of multiple linear regression analyses exploring the effects of age and sex on spatiotemporal gait parameters, adjusted for BMI and test center are shown in Table 4. Increasing age was associated with significant lower performance for mean values and CoV for all gait parameters, except for the mean value of stride width (*P* = 0.861) and CoV of double support time (*P* = 0.186). Women demonstrated lower mean values for all temporal gait parameters compared to men, except for the mean value of double support time (*P* = 0.059) CoV of spatial parameters were significantly greater in women compared to men. In addition, both mean and CoV of stride velocity were significantly worst with increasing age in women.

**Table 4:** Multiple linear regression showing the association of spatiotemporal gait parameters (dependent variables) with age and sex (independent variables) adjusted for body mass index and test center among participants (*n* = 954).

Spatiotemporal gait parameters^*^ (Dependent variables)	**Independent variables**					
	**Age**			**Sex**		
	**β**	**[95%CI]**	***P*-value**	**β**	**[95%CI]**	***P*-value**
**STRIDE TIME**						
Mean value (ms)	3.14	[1.55; 4.73]	**<0.001**	−50.62	[−65.85; −35.38]	**<0.001**
CoV (%)	0.04	[0.02; −0.05]	**<0.001**	00.13	[−0.00; 0.25]	0.056
**SWING TIME**						
Mean value (ms)	−0.52	[−1.03; −0.00]	**0.049**	−21.69	[−26.62; 16.76]	**<0.001**
CoV (%)	0.10	[0.07; 0.12]	**<0.001**	−0.02	[0-0.25; 0.21]	0.880
**STANCE TIME**						
Mean value (ms)	3.51	[2.34; 4.69]	**<0.001**	−31.11	[−42.38; −19.83]	**<0.001**
CoV (%)	0.03	[0.01; 0.05]	**0.004**	0.14	[−0.04; 0.31]	0.122
**SINGLE SUPPORT TIME**						
Mean value (ms)	−0.59	[−1.10; −0.09]	**0.021**	−22.66	[−27.50; −17.82]	**<0.001**
CoV (%)	0.10	[0.08; 0.13]	**<0.001**	−0.00	[−0.23; 0.22]	0.992
**DOUBLE SUPPORT TIME**						
Mean value (ms)	−4.03	[3.14; 4.92]	**<0.001**	−8.22	[−16.73; 0.30]	0.059
CoV (%)	−0.03	[−0.06; 0.01]	0.186	0.34	[−0.01; 0.70]	0.057
**STRIDE LENGTH**						
Mean value (cm)	−1.49	[−1.68; −1.29]	**<0.001**	−14.48	[−16.34; 12.62]	**<0.001**
CoV (%)	0.07	[0.06; 0.09]	**<0.001**	0.22	[0.08; 0.36]	**0.002**
**STRIDE WIDTH**						
Mean value (cm)	0.00	[−0.04; −0.04]	0.861	−0.95	[−1.33; −0.57]	**<0.001**
CoV (%)	0.77	[0.11; 1.44]	**0.023**	8.09	[1.71; 14.47]	**0.013**
**STRIDE VELOCITY**						
Mean value (cm/s)	−1.47	[−1.75; −1.20]	**<0.001**	−2.62	[−5.23; −0.01]	**0.049**
CoV (%)	0.05	[0.03; 0.07]	**<0.001**	0.27	[0.08; 0.46]	**0.005**

ms, millisecond; s, second; cm, centimeter; CoV, coefficient of variation; CI, confidence interval; β, coefficient of regression corresponding to a decrease or increase in value of gait parameters;

* *Used as dependent variable in the multiple linear regression. P-value significant (i.e., < 0.05) indicated in bold; each linear Model is adjusted for Body mass index and test center*.

## Discussion

Standardized systematic assessment of three categories of information, which included demographics, clinical features and gait characteristics were selected for the development of gait assessment guidelines. Two complementary sets of guidelines have been proposed: a minimal data set and a full data set. Concerning the quantitative reference values, we observed lower values in several spatiotemporal gait parameters with age as well as differences between men and women. Age had a negative effect on mean values and CoV, while sex was mainly associated with mean values. Stride velocity parameters were affected both by age and sex.

Our study provides quantitative normative values for widely used and clinically relevant spatiotemporal gait parameters. Compared to previous studies on this topic, the strategy of recruiting participants through an intercontinental initiative provides access to probably the highest number of participants involved in a study exploring reference values until now. Furthermore, we chose to select “very healthy” older participants to avoid any interaction with morbidities or cognitive impairments that can affect gait performance. Previous studies have controlled for the potential effects of morbidities using statistical analysis (Oberg et al., 1993; Oh-Park et al., 2010; Bohannon and Williams Andrews, 2011; Hollman et al., 2011; Hass et al., 2012). However, it has recently been suggested that the strategy of statistical adjustment may be limited and does not take into consideration the complex interplay and potential effects of morbidities (Kressig et al., 2006; Byrne and Pachana, 2011; Montero-Odasso et al., 2012). For instance, a recent study reported the results of the independent and combined effects of impairments of muscle strength, distance vision, lower-limb proprioception, and cognition on gait performance using pTUG and iTUG (Byrne and Pachana, 2011). It was shown that cognitive impairment, considered either separately or in combination with any other subsystem decline, notably muscle strength, was strongly associated with decreased performance on the pTUG and delta TUG scores. In contrast, lower-limb proprioceptive impairment was associated with worse performance (i.e., lower) on the iTUG. The subsystem's impairment has been associated with worse (i.e., greater) delta TUG scores; the highest impact being reported when combining muscle strength and cognition. In our study, all participants were free of morbidities, and thus provided the opportunity to report real normative quantitative reference values by age category from 65 to 85 years and above. The decline in gait performance with age is consistent with the literature and supports the validity of the reported values.

Some limitations, however, need to be acknowledged. First, the number of participants in the 85 and over age category was low, probably because healthy individuals only represent a low percentage of this age group. More effort needs to be made to explore this population, as they currently represent the fastest growing age group in many countries and have the highest prevalence and incidence of gait disorders (American Geriatrics Society and British Geriatrics Society and American Academy of Orthopedic Surgeons Panel on Falls Prevention, 2001; Panel on Prevention of Falls in Older Persons and American Geriatrics Society and British Geriatrics Society, 2011). Second, because this initiative merged data from clinical and research centers in different countries and different clinical settings, assessment was not strictly uniform even if the same procedures and equipment were used.

## Conclusions

The past decade has been characterized by an acceleration of knowledge in medicine and science, particularly in the area of neuroscience. Considerable efforts have been (and continue to be) made in developing accessible and practical technology-based assessment tools aiming at providing accurate measurements of spatiotemporal gait parameters. These advances challenge researchers and clinicians, pushing them to develop new ways of thinking and working. Currently, new opportunities exist as the result of working as part of an internationally structured consortium. The GOOD initiative (Allali et al., 2016) underscores the fact that there is still a lot of work to do, but significant progress has been made and the future is optimistic with respect to the development of the Biomathics and Canadian Gait Consortiums. This work represents an important first step in the development of guidelines for clinical and spatiotemporal gait analysis based on the recorded footfalls in laboratory setting and the definition of quantitative reference values in healthy older adults. These guidelines facilitate the ability to work together and think broadly and effectively in the field of gait disorders and aging.

## Author contributions

Study concept and design: OB, GA and JH; acquisition of data: OB, JV, JS, RK, CL, MC, VS, AD, and JH; analysis and interpretation of data: OB, GA, and JH; drafting of the manuscript: OB, GA, CL, and JH; critical revision of the manuscript for important intellectual content: HS, JV, SGu, JS, RK, JB, TS, CL, SGr, LB, TL, VC, MC, VS, GL, AD, RS, GD, and RC; obtained funding: OB, JV, and JH; statistical expertise: OB; administrative, technical, or material support: OB and JH; study supervision: OB and JH. All the authors have participated in the research reported, have seen and approved the final version of the manuscript, and have agreed to be an author of the paper.

### Conflict of interest statement

The authors declare that the research was conducted in the absence of any commercial or financial relationships that could be construed as a potential conflict of interest.
